# Periprosthetic Femoral Fractures-Beyond B2

**DOI:** 10.5435/JAAOSGlobal-D-23-00135

**Published:** 2024-08-06

**Authors:** Chika Edward Uzoigwe, Arun Thor Watts, Praise Briggs, Tom Symes

**Affiliations:** From the Harcourt House Sheffield, UK (Mr. Uzoigwe); Hull Royal Infirmary, Hull, UK (Mr. Watts, Mr. Briggs, and Mr. Symes).

## Abstract

The proliferation of hip arthroplasty has seen concomitant increases in periprosthetic femoral fractures (PFFs). The most common pattern involves fracture at the level of a loose prosthesis (B2). B2 PFFs have a unique mechanopathogenesis linked to the tendency of polished taper-slip cemented stems to subside in the cement. Such stems carry a much higher PFF risk than other cemented designs. Mega-data, consistent across national registries, suggest that increasing application of the taper-slip principle has resulted in the emergence of highly polished, very low friction cemented prostheses. These have the propensity to migrate within the cement, increasing B2 PFF risk. This would explain the strong association between cobalt-chromium stems and PFF. Is PFF the mode of failure of polished taper-slip stems rather than aseptic loosening? Established wisdom teaches that B2 PFFs should be managed with revision surgery. There is a large body of new evidence that, in certain instances, fixation results in outcomes at least equivalent to revision arthroplasty, with shorter surgical time, decreased transfusion requirements, and lower dislocation risk. This is so in B2 PFFs around cemented polished taper-slip stems with an intact bone-cement interface. We outline advances in understanding of B2 PFF with special reference to mechanopathogenesis and indications for fixation.

Increasing patient longevity has led to a precipitous increase in the incidence of periprosthetic femoral fractures (PFFs). They are projected to become a sizeable component of the fragility fracture epidemic.^1^ According to the UK National Mayo Clinic Joint Replacement Database, the percentage contribution of periprosthetic fractures to the number of revision hip surgeries has increased from 9% in 2012 to 22% in 2020 (Figure 1; https://www.njrcentre.org.uk/). The year 2020 saw the introduction of mandatory recording and financial incentivization of the care of patients with PFF, through the UK National Hip Fracture Database (https://www.nhfd.co.uk/).

**Figure 1:**
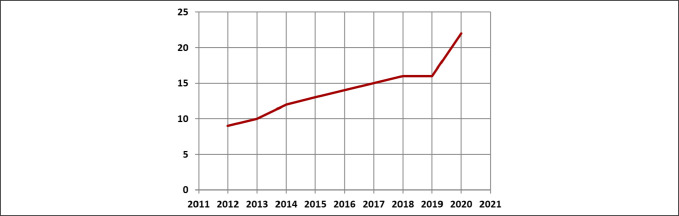
Plot demonstrating the percentage of revisions in the UK National Mayo Clinic Joint Replacement Database due to periprosthetic fracture.

Vancouver B2 describes a periprosthetic femoral fracture (B2 PFF) at the level of a loose prosthesis, but with adequate bone stock. These fractures have courted the most controversy (Table 1).^4^ Established wisdom teaches that this fracture configuration necessitates revision.^5^ However, in recent years, an increasing body of strong evidence has started to emerge showing that, in certain instances, fixation of B2 PFF leads to outcomes at least equivalent to and, according to some parameters, superior to revision arthroplasty.^6,7^ Unselective use of revision surgery for all periprosthetic fractures may expose patients to additional risks with no clear benefit. Here, we collate the recent literature regarding the pathomechanics and management of postoperative Vancouver B2 fractures, with special reference to fracture fixation in cemented and noncemented stems.

**Table 1:** Vancouver Classification and Modification

Vancouver			Fracture Position
A			Above prosthesis
	AG		At the greater trochanter
	AL		At the lesser trochanter
B			At the level of prosthesis
	B1		Well-fixed stem
	B2		Loose stem
		B2W	Loose stem—bone-cement interface intact
		B2L	Loose stem—bone-cement interface disrupted
		B2.1	Loose stem—fracture at 1 Gruen zone^2^
		B2.2	Loose stem—fracture at more than 1 Gruen zone^2^
		B2clam	Fracture of the lesser trochanter—lateral cortex intact^3^
		B2reverseclam	Fracture involving the greater trochanter—medial cortex intact^3^
		B2burst	Comminution at stem—medial and lateral cortex disruption
		B2spiral	
	B3		Loose stem + poor bone stock
C			Below prosthesis

## Mechanopathogenesis of B2 Fractures: The Stem of the Problem

The UK National Hip Fracture Database 2023 report identified Vancouver B PFFs to be the most common fracture pattern, comprising 65.7% of the 2,986 reported cases of PFF (https://www.nhfd.co.uk/2023report). Furthermore, B2 PFFs are the most common subclass, constituting 39% of all postoperative periprosthetic hip fractures, in the large diverse 12,900-patient strong meta-analysis by Deng et al.^8^

### Stem Designs

**Figure 2:**
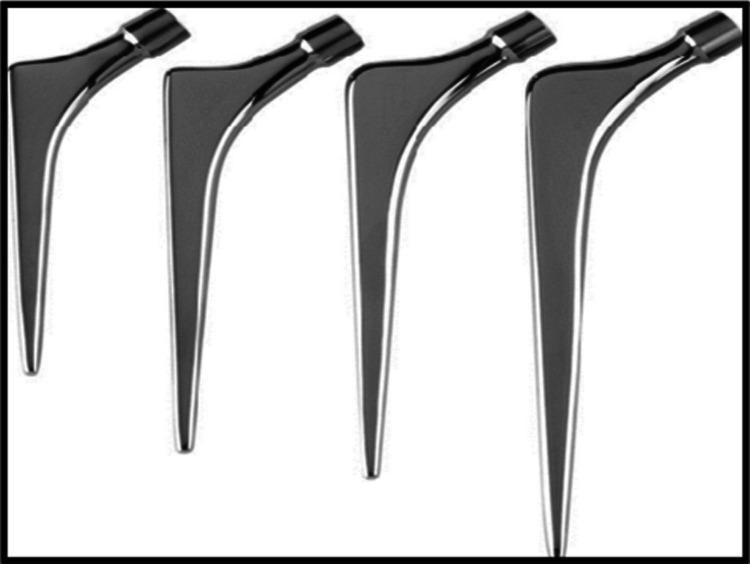
Image showing the classical polished taper-slip stem. The Exeter stem from https://www.stryker.com/tn/en/joint-replacement/products/exeter/index-eu.html.

**Figure 3:**
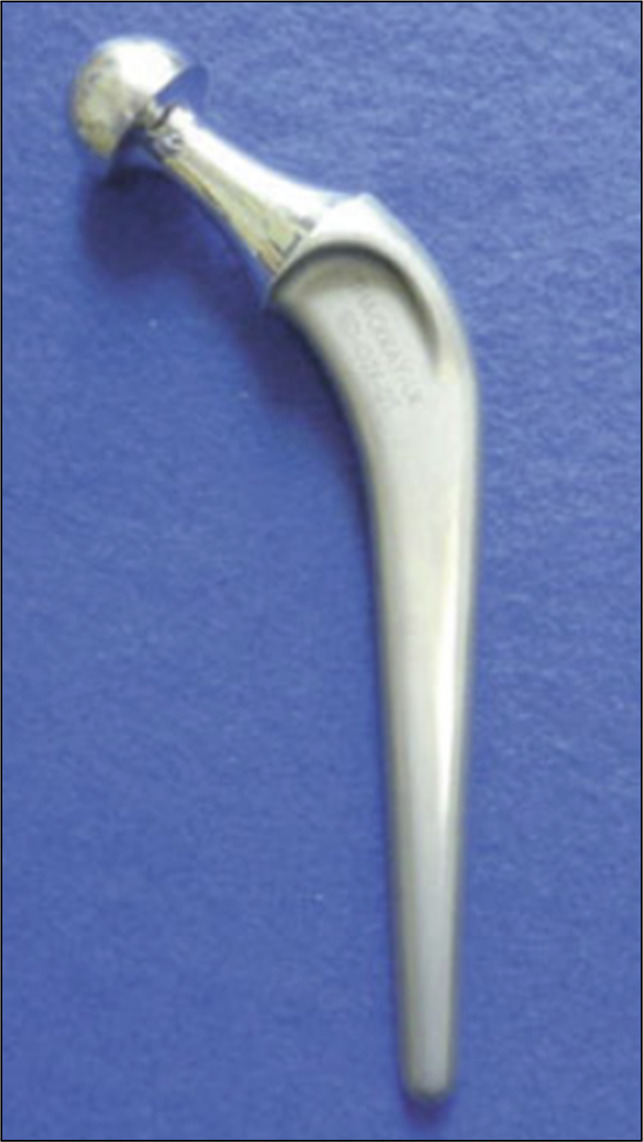
Image showing an example composite beam stem (the Charnley) fromfile:///C:/Users/ch191842/Downloads/Biomechanical_and_Tribological_Aspects_of_Orthopae.pdf.

Stems have different properties affecting periprosthetic femoral fracture risk. Cemented/noncemented is the first relevant dichotomy. Among cemented stems exist the polished taper-slip (Figure 2) and composite beam (Figure 3) stems. The former class include the Exeter (steel), CPT (cobalt chromium), and CPS (steel). The Charnley, contrarily, is a composite beam stem.

### Cemented Stems: The Polished Taper-Slip Stem

Evidence suggests that B2 fractures have a unique pathogenesis in which stem design is strongly implicated (Supplemental Table file). The polished taper-slip stem (also known as force-closed/Exeter-type) subsides in the cement mantle at the stem-cement interface. The cement-bone interface is immobile. This stem subsidence generates radial forces and hoop stresses^9^ in the cement, strengthening the cement-bone bond. However, it may increase the risk of Vancouver B2 periprosthetic femoral fracture after trauma.^10^ Phillips et al suggested that the polished stem acts as an axe, cleaving the cement and bone envelope.^11^ In a biomechanical study involving the polished taper-slip stems, Exeter, CPT, and CPS, an axial load and rotational force generated exclusively B2 PFFs.^11^

#### Stem Size

Other factors also contribute to risk. Placing a smaller cemented stem size than the femur can accommodate has increased PFF risk in one small study.^12^ Here, the authors compared implanted stem size with optimum stem size as determined by templating. A smaller stem increases the acuteness of the taper while exposing the cement-bone conjugate to higher pressures. A subsized stem is essentially akin to a sharper “axe.” Templating may, therefore, mitigate risk.

#### Sex

Studies looking at large registry data have found that among cemented prostheses, Vancouver B fractures are more common in men while C fractures (distal to the stem) are more common in women.^13^ This suggests different pathomechanisms. Osteoporotic fractures are more common in women; hence, Vancouver C PFFs may be a fragility-type fracture. The higher incidence of B fractures in men supports the “axe” model because men have a higher mass and thus exert a higher axial/rotational load.

### Cemented Stems: Polished Taper-Slip Versus Composite Beam Stem

In contrast to the polished taper-slip stem, the “composite beam” (also known as shape-closed/Charnley style) cemented prosthesis tends not subside to as great a degree. Its longevity depends on a strong cement-prosthesis interface.^14^ For this reason, it is termed “composite beam” because the prosthesis-cement dyad moves as one. They are also termed shape-closed because the stem-cement fixation is achieved through tessellation of the surfaces because of the roughness of the stem. Tessellation describes where surfaces fit together with no spaces.

Consistent with the “axe” paradigm, the national registry data show that polished taper-slip stems are associated with a much higher risk of B PFF than the composite beam. Chatziagorou et al compared over 28,100 Exeter (polished taper) and 52,500 Lubinus SPII (composite beam) stems in the Swedish registry with a median follow-up of 5.6 years (range 2-11).^6^ The risk of Vancouver B PFF was 10 times higher in the Exeter while there was no difference in Vancouver C risk between the two. This finding is consistent across registries. Using data from the Norwegian Hip Fracture Register, Kristensen et al^15^ compared the rate of surgery for periprosthetic femoral fracture in the Exeter V40 and Lubinus bipolar hemiarthroplasties. The adjusted risk of revision surgery for PFF was 10-fold higher for the Exeter V40 bipolar stem than for Lubinus. Similarly, the Nordic registry reported a 5-fold increase in risk of revision for PFF for the Exeter compared with Lubinus at 2 years.^16^ The advantage of the Norwegian and Swedish registries is that, unlike the UK registries, they report not only periprosthetic femoral fracture undergoing revision but those where fixation is performed.

### Cemented Stems: Too Polished?

Polished taper-slip stem subsidence appears literally to be a double-edged axe: protective against aseptic loosening but increasing the periprosthetic femoral fracture risk—the greater the subsidence tendency, the greater the periprosthetic femoral fracture risk.

Palan et al,^17^ on analysis of the UK National Mayo Clinic Joint Replacement Database, reported that the CPT (polished taper-slip stem) had a revision rate for PFF that was almost 4 times higher than that for the Exeter. The rate for the C-stem was equivalent to that for the Exeter. The revision rate for the Charnley (composite beam) stem was less than half that of the Exeter. Periprosthetic femoral fractures undergoing fixation are not included in the UK registry. This problem is circumvented in the Nordic registries where both fixations and revisions are included. Kristensen, using the Norwegian registry, observed a 3.2-fold higher periprosthetic femoral fracture rate in the CPT hemiarthroplasty stem compared with the Exeter hemiarthroplasty stem.^15^

Lamb et al looked at the UK National Mayo Clinic Joint Replacement Database for periprosthetic femoral fracture risk factors, *specifically in polished taper-slip stems*. They found cobalt-chromium stems strongly associated with periprosthetic femoral fracture.^18^ The use of cobalt-chromium stems increased the risk of PFF revision nearly 7-fold (95% CI: 3-15) compared with stainless steel stems. Implantation of the cobalt-chromium stem with low viscosity cement was associated with a 23-fold increase in adjusted revision risk of PFF (95% CI: 10-53) compared with the stainless steel stems. The common thread in the works of Palan and Lamb is that CPT is composed of cobalt-chromium and is by far the most commonly used prosthesis of this material in the UK registry. Exeter and C-stem are composed of stainless steel.

The reason for the increased periprosthetic femoral fracture risk attendant to CPT stems had hitherto remained unclear. It has been attributed to the CPT geometry. However, Lamb et al adjusted for the potential confounding effect of stem stereo geometry.^16^ Others have attributed it to increased wear of the cobalt-chromium stem. However, cobalt-chromium stems are harder than the Exeter stainless steel alloys. A likely explanation for the higher PFF rate in cobalt-chromium polished taper-slip stems has emerged with biomechanical studies. The CPT stem and cobalt-chromium polished taper-slip stem, as a class, have a number of features that markedly increase their proclivity to subside in the stem. It is this predisposition to subsidence that may increase the risk of PFF.

To facilitate subsidence, taper-slip stems are polished to reduce asperities. These are minute irregularities of the surface with peaks and troughs. The peaks are termed the asperities. Roughness (Ra) is the average distance from the asperity peaks to nadirs on a solid surface. Manufacturers' data suggest that the CPT stem is more polished than the Exeter stem. Roughness of the Exeter is reported as less than 0.05 µm. Roughness of the C-stem is reported as 0.02 to 0.17 µm while that of the CPT stem is 0.025 to 0.05 µm.^19^ More markedly, in a biomechanical study, Hirata et al^20^ reported that cobalt-chromium showed lower adhesiveness than stainless steel and thus would exert weaker molecular adherent forces to cement when compared with stainless steel at identical material roughness. Furthermore, they reported that at a surface roughness of 0.1 µm and less, cobalt-chromium showed a lower coefficient friction with cement compared with stainless steel of identical roughness. The difference was more pronounced when medium-viscosity cement was used compared with high viscosity. This biomechanically corroborates the findings of the Lamb group and the strong association between the cobalt-chromium stems and periprosthetic femoral fracture, necessitating revision surgery.

Tsuda et al, using cobalt-chromium and stainless steel stems of roughness less than 0.2 µm, found that the cobalt-chromium prosthesis underwent markedly greater subsidence than the stainless steel prosthesis.^21^ A recent meta-analysis reported a three-fold higher subsidence rate for the CPT (cobalt-chromium) stem at 0.37 mm/year compared with the Exeter at 0.10 to 0.16 mm/yr.^22^

The Exeter Trauma Stem monoblock hemiarthroplasty used in hip fracture has a much higher surface roughness at 0.24 µm compared with the Exeter V40 at 0.025 µm.^23^ Evidence suggests the Exeter Trauma Stem has a lower periprosthetic femoral fracture rate than the V40.^24^ It is unclear whether this feature of the Exeter Trauma Stem at creation in 2002 was by design or serendipity. However, it was inspired, potentially reducing PFF risk.

## Management of Vancouver B2 Periprosthetic Fractures

### Historical Consensus

The Vancouver paradigm conceptually first appeared in the literature in 1981. Dr Joyce Johansson et al described “*Fracture of the Ipsilateral Femur in Patients with Total Hip Replacement.*” The Vancouver classification was first explicitly articulated in 1995 by Dunn and Masri, working in Vancouver.^25^ On the basis of this classification, Lewallen and Berry^26^ suggested revision arthroplasty for B2 fractures in 1998. This has remained orthopaedic orthodoxy for over 20 years. However, a panoply of papers in 2020s challenge this especially in the context of polished taper-slip stems (Supplemental Table file, http://links.lww.com/JG9/A348).

A review of 130 members of the European Hip Society found that 28% would perform open reduction and internal fixation for Vancouver B2 fractures for noncemented periprosthetic femoral fracture.^27^ The 2020 National Hip Fracture Database report, the first audit of its kind, recorded that in the United Kingdom, 50.5% of Vancouver B fractures (B1/B2/B3) were treated with open reduction and internal fixation. In 2024, this decreased to 37%.

### Outcomes of Fixation Vancouver B2

#### Cemented Stems: Polished Taper-Slip Stem

Slullitel et al^28^ compared Exeter PFFs in low-demand patients. They explored the outcomes of three cohorts: 47 B1 and 27 B2 fractures treated with fixation and 38 B2 fractures treated with revision. The decision for osteosynthesis over revision was made on the basis of an intact bone-cement interface (termed B2W). The integrity of the bone-cement junction was determined intraoperatively, by which it can be definitively seen where the bone and cement remain well bonded compared with regions of disengagement. They found that implant survival, adverse events, and mortality rate were the same for all three groups. They concluded that, where the bone-cement interface is intact and the patient elderly/low-demand, B2 PFF around polished taper-slip stems can be treated with internal fixation.

Powell-Brown^29^ went further to compare revision and fixation in 50 Exeter Vancouver B2 PFF. Internal fixation resulted in lower revision rates, a higher 5-year implant survival, and lower transfusion requirements, especially where the bone-cement interface was intact. The authors suggested that where the bone-cement interface is intact and anatomical reduction achievable, all B2 fractures around polished taper-slip stems can be managed with open reduction and internal fixation. If reduction is nonanatomical, union occurs by secondary bone healing, which requires movement at the fracture and thereby potentially accelerating stem loosening.

#### Noncemented Stems

A similar epiphany was emerging among surgeons using noncemented prostheses. The authors highlighted the importance of anatomical reduction for successful outcomes of B2 noncemented PFF treated with fixation. Again in 2021, Martinov reported an analysis of 81 B2 PFFs around noncemented stems. Fifty-four were treated with fixation and 27 with revision, with a median follow-up of 5 years.^30^ No cases of failure were found in the fixation cohort. The groups were radiographically equivalent. This is notwithstanding the fact that in some cases, rudimentary techniques were used such as isolated cerclage wires.

**Figure 4:**
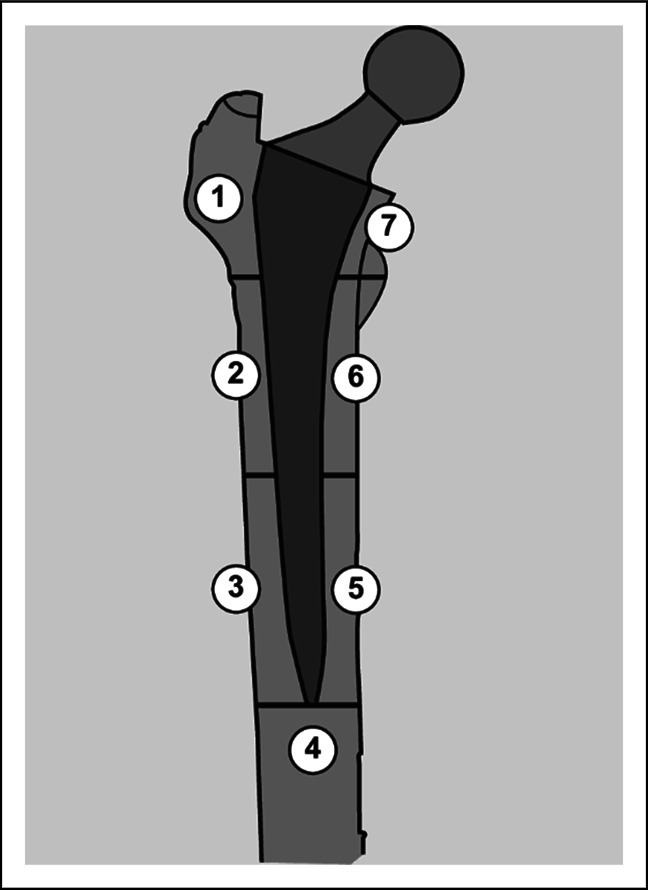
Illustration of Gruen zones from Cavalli L, Brandi ML. Periprosthetic bone loss: diagnostic and therapeutic approaches. F1000Res. 2014 Jun 17; 2:266. doi: 10.12688/f1000research.2-266.v2. PMID: 25642325; PMCID: PMC4304431.

Other authors began to consider the effect of the location of the fracture within the proximal femur. The proximal femur around the prosthesis can be divided into seven zones, known as Gruen zones (Figure 4). Gonzalez-Martin, also published in 2021, found that in cases where the B2 noncemented periprosthetic femoral fracture involved only one Gruen zone, favorable outcomes were achieved with fracture fixation. In their study, there were no implant complications in 15 Vancouver B2 fractures around noncemented stems treated with osteosynthesis where the fracture involved only one Gruen zone. However, there were five implant-related adverse events in the 15 cases where two or more Gruen zones involved. Gonzalez-Martin et al also strongly emphasized the importance of anatomical reduction in achieving favorable outcomes in the noncemented B2 PFF cohort.

Spina et al^31^ reported good or excellent radiographic and clinical outcomes in B2 PFFs around a noncemented stem treated with osteosynthesis. They found that stem shape played a role. Straight stems are almost perfectly straight. Anatomical stems mirror the contour of the proximal femur which is bowed. Straight stems had more favorable outcomes after fixation for B2 PFF. Better outcomes were also achieved where the fracture did not extend beyond the tip of the prosthesis and the tip of the stem remained contained in the femoral shaft. The authors postulated that the straight stem design allowed re-osseointegration after fracture fixation because of areas of stem-bone fixation unaffected by fracture or after unintentional noncemented stem subsidence to the new site. Success of B2 periprosthetic femoral fracture fixation around noncemented stems necessarily relies on re-osseointegration between bone and the stem. Pilliar showed that micromotion of less than 20 µm is conducive to this process. Between 20 µm and 150 µm, there is combination of fibrous and bone tissue at the interface. Above 150 µm of micromotion, fibrous tissue predominates.^32^

## Fixation Versus Revision for Vancouver B2 Periprosthetic Femoral Fracture

Recent evidence has consistently reported shorter surgical time and reduced transfusion requirements for fixation over revision surgery.^26^ Four large meta-analyses and systematic reviews published in 2020, 2021, 2022, and 2023 support fixation as a therapeutic option for B2 periprosthetic femoral fracture.^6,7,33,34^ Haider et al in their meta-analysis included over 2,500 patients with B2 and B3 periprosthetic femoral fractures around both cemented and noncemented stems. In a subgroup analysis of Vancouver B2 fractures, they observed no difference between those treated with revision and those treated with osteosynthesis regarding complications, revision, mortality, union rates, Harris Hip Score, and Parker Mobility Score.^6^ In 2022, Lewis et al in their meta-analysis looked at over 1,620 patients with B2 fractures in noncemented and cemented prostheses. They found that those treated with fixation had equivalent complication and revision rates compared with those treated with revision arthroplasty. The dislocation rate was lower in cases where fixation was performed than where revision surgery was undertaken (1.3% vs. 4.8%). Stoffel et al. conducted a systematic review of 14 studies. They compared fixation and revision surgery for B2 PFFs. The authors found five studies in favor of fixation, and the others were indeterminate. The authors concluded that recent data suggest that surgical fixations result in favorable outcomes in cemented prostheses with taper-slip stems with an intact cement mantle. In the case of noncemented stems, anatomical reduction is the most important factor in achieving positive outcomes.

One limitation of the meta-analyses is that they generally do not distinguish between cemented and noncemented stems nor between polished taper-slip stem and composite beam (Charnley type) stems. Additional inclusion of historical data can be problematic.

### Unanswered Questions: Cemented Composite Beam

The success of internal fixation in cases of polished taper-slip stem PFF is predicated on the dynamic cement-stem interface. It is unclear whether the same polished taper-slip stem periprosthetic femoral fracture principles can be applied to the composite beam stems. The stability of this prosthesis is contingent on “physical engagement” between the stem and cement. Given that even composite beam stems subside minimally,^14^ it is likely that stability relies on stem cement friction rather than perfect interdigitation between cement-stem asperities. The question remains as to whether the stem-cement interface can be reconstituted to sufficient stability with fixation surgery in composite beam stems.

### Cement in Cement Revision

Successful internal fixation of polished taper-slip stem PFF depends on the ability to achieve anatomical reduction and subsequently stable stem. Where these criteria are not met, revision is indicated. However, for the polished taper-slip stem, the novel technique of cement-in-cement revision also remains an option. Standard revision involves removal of the prosthesis and the cement. Cement-in-cement revision involves removal of the prosthesis alone. It is performed if the cement is still well fixed to the bone. The fracture is fixed. The cement mantle is preserved. A new prosthesis is then cemented in place with fresh cement, creating a cement-in-cement revision. A number of authors have reported very good results from this technique. The success of the method is contingent on the bone-cement complex integrity. In 2021, Maggs et al^35^ examined 87 PFFs in cemented prostheses. They found that the polished taper-slip stem tended to fracture in the B2W pattern, hence at the level of the prosthesis with well-fixed cement-bone junction mantle. However, composite beam prostheses tended to exhibit the Vancouver B2L (loose bone-cement interface) PFF configuration. In their study, there were 47 Vancouver B2W (well-fixed bone-cement interface) fractures. These were treated with cement-in-cement revision. 43 of 47 cases proceeded to union.

Klassan et al compared 70 noncemented long stem revisions with 30 cement-in-cement revisions for patients who had suffered Vancouver B2 PFF but with an intact cement-bone interface (B2W). The results between the groups were equivalent. However, surgical time was shorter with the cement-in-cement procedure. No difference was observed in in-hospital stay, surgical complications, readmissions, patient survival, or implant longevity.^36^ The cement-in-cement arthroplasty stem may be either a smaller stem or a long stem, both showing favorable outcomes.^37^

## Treatment Algorithm

**Figure 5:**
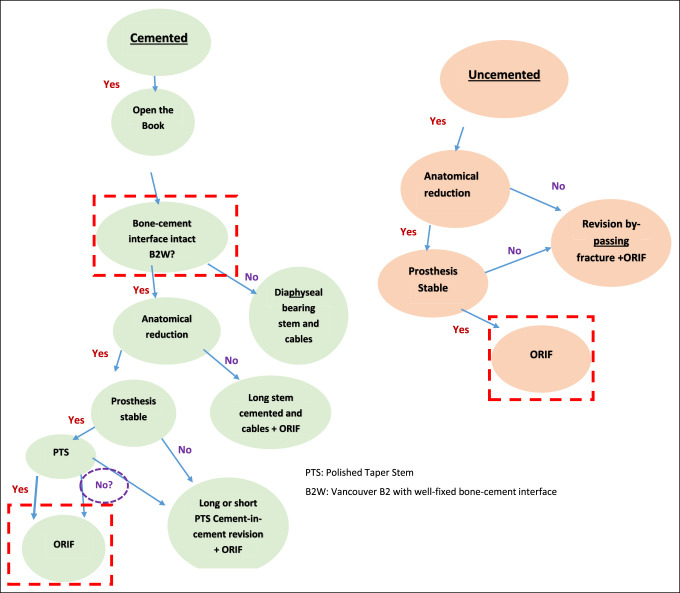
Flow diagram showing management algorithm of periprosthetic fractures. PTS = polished taper stem, B2W = Vancouver B2 with well-fixed bone-cement interface.

The evidence allows the formulation of an algorithm for the management of B2^39^ (Figure 5).

### Cemented Prosthesis

In the case of cemented prostheses, access the fracture (open the book). If the bone-cement integrity is preserved with no/minimal bone loss, see whether anatomical reduction is achievable. Computerized tomography may give an indication as to whether anatomical reduction is possible. However, this is determined definitively intraoperatively. Where there are two or three fragments, then anatomical reduction is more probable. However, where there is extensive communition, this is less likely possible. Similarly, where the stem tip has dislocated from the cement restrictor, anatomical reduction is less likely to be successful. Temporary fixation is maintained with Hey Groves, collinear clamps, and/or cables or Fibretape. Prosthesis stability is assessed after reduction. This is achieved by determining whether the prosthesis visibly toggles within the cement mantle or subsides on manual axial pressure. If the triad of interface integrity, fracture reducibility, and stem stability is satisfied, open reduction is a valid option. Osteosynthesis plates specifically designed for periprosthetic fractures with diverse fixation modalities should be considered. Such implants include the possibility of polyaxial screws, locking and nonlocking options, and the facility to pass cables through the plate and uncinated (hooked) modular attachments to engage to the greater trochanter. The surgeon should consider engaging the length of the femur with plate fixation to prevent future periprosthetic fracture^4,5.^ This is achievable by minimally invasive techniques.

In instances where the bone-cement interface is well fixed with no bone loss but anatomical reduction is not achievable and/or the prosthesis is unstable, consider short stem cement-in-cement revision in combination with internal fixation or long stem cement-in-cement revision with cables. Cebotome can be used to make the canal more capacious. This is a very high-speed, low-torque burr for removing cement.

Where there is extensive comminution or proximal bone loss or the cement is loose, it is ideally necessary to revise the stem to a modular diaphyseal bearing noncemented stem.^4,38^ This may not be viable in frail patients with limited physiological reserve. In this context, proximal femoral placement may be preferable.^39^

Guidance is more difficult in cases of cemented composite beam stems. It is unclear whether durable fixation is achievable in this context. Some argue that B2 PFF around such prostheses mandates revision surgery, which can be cement-in-cement. Hence, where composited beam stems are involved, extraneous factors such as the patient comorbid burden and functional status become notable relevant considerations.

### Noncemented Prosthesis

In the case of noncemented prostheses, a similar model is followed. Assess whether the prosthesis is well fixed distally. This is done intraoperatively. The surgeon assesses whether the prosthesis is rigidly fixed to the bone distal to the fracture site or if it can be readily removed. It the prosthesis is well fixed distally, consider proceeding to fixation. If the stem is loose/unstable, consider revision surgery to a modular diaphyseal bearing noncemented stem with cable supplementation.^4,38^ Questions still remain as to whether a robust and longevous fixation is ever achievable with fixation alone after B2 noncemented PFF. Patient factors are important in the decision-making process.

Great strides are currently being made to determine how best to treat periprosthetic femoral fracture (https://clinicaltrials.gov/ct2/show/NCT03378557). Aseptic loosening has classically been used as the index event of stem failure in stem survivorship analyses. This may not be appropriate. Recent data suggest that the Exeter has a 10-year aseptic loosening rate of 0% but a Vancouver B2 periprosthetic femoral fracture rate of 2%.^40^ Indeed, PFF may be the mode of failure of the polished taper-slip stem rather than aseptic loosening. The total hip arthroplasty was the operation of the 20th century. The periprosthetic fracture promises to be the fracture of the 21st century. Comprehensive data are required on the care of this vulnerable cohort of patients.

## Supplementary Material
