# Supplementary material for: Periprosthetic Femoral Fractures-Beyond B2
Source: J Am Acad Orthop Surg Glob Res Rev. 2024 Aug 6;8(8):e23.00135. doi: 10.5435/JAAOSGlobal-D-23-00135 (PMC11309721; doi:10.5435/JAAOSGlobal-D-23-00135)
Supplement: Supplementary file 1 [file jagrr-8-e23.00135-s001.docx]

| **Study** | **year** | **Location/**  **Study type** | **Key finding** | **Level of evidence** |
| --- | --- | --- | --- | --- |
| **Higher Risk of PPF in with PTS vs Other cemented stems** | | | | |
| *Mohammed J et al.*  Reduced periprosthetic fracture rate when changing from a tapered polished stem to an anatomical stem for cemented hip arthroplasty: an observational prospective cohort study with a follow-up of 2 years.  Acta Orthop. 2019 Oct;90(5):427-432. | 2019 | Stockholm, Sweden | Transition from PTS (CPT) to Anatomic cemented stem (Lubinus) for total and hemarthroplasty reduced PPF rate from 3.3% to 0.4%. Use of the anatomic stem reduced risk by a factor of 0.1 | II |
| *Brodén C et al.*  High risk of early periprosthetic fractures after primary hip arthroplasty in elderly patients using a cemented, tapered, polished stem. Acta Orthop. 2015 Apr;86(2):169-74. | 2015 | Stockhold, Sweden | 3.3% 2-year PPF risk for CPT stem hip arthroplasty performed for osteoarthritis or femoral neck fracture | II |
| *Thien TM et al.*  Periprosthetic femoral fracture within two years after total hip replacement: analysis of 437,629 operations in the nordic arthroplasty register association database.  J Bone Joint Surg Am. 2014 Oct 1;96(19):e167 | 2014 | Nordic Arthroplasty Register (Denmark, Sweden, Norway) | Exeter (PTS) has a 5 fold increase in risk for PFF than Lubinus (anatomic stem)  (95% CI: 3.3-7.7) | II |
| *Kristensen TB, et al*  More reoperations for periprosthetic fracture after cemented hemiarthroplasty with polished taper-slip stems than after anatomical and straight stems in the treatment of hip fractures: a study from the Norwegian Hip Fracture Register 2005 to 2016.  Bone Joint J. 2018 Dec;100-B(12):1565-1571. | 2018 | Norwegian Hip Fracture register | Use of composite beam (Charnley stem) for hemiarthroplasty in hip fracture reduced risk of periprosthetic fracture by a factor or 0.64 compared to Exeter (PTS) | II |
| *Mellner C et al.*  Increased risk for postoperative periprosthetic fracture in hip fracture patients with the Exeter stem than the anatomic SP2 Lubinus stem.  Eur J Trauma Emerg Surg. 2021 Jun;47(3):803-809. | 2021 | Sweden  Multi-centre | At medial follow-up 47 months PPF risk was 2.3% for Exeter PTS and 0.7% for Lubinus Anatomic Stem (p<0.0001) in hip fracture patients undergoing total hip arthroplasty. Using the Exeter PTS increased PPF risk by factor of 5.4 (956% CI: 2.4-12.5) | III |
| *Scott T et al.*  Polished, collarless, tapered, cemented stems for primary hip arthroplasty may exhibit high rate of periprosthetic fracture at short-term follow-up.  J Arthroplasty 2018;33:1120-1125. | 2018 | New York, USA | CTP (PTS) stem had a 2.2% PPF risk compared to 0.25% for composite beam | III |
| **Higher Risk with higher amongst Cobalt Chromium stems CPT stems** | | | | |
| *Palan J, et al*  The influence of cemented femoral stem choice on the incidence of revision for periprosthetic fracture after primary total hip arthroplasty: an analysis of national joint registry data.  Bone Joint J. 2016 Oct;98-B(10):1347-1354. | 2016 | UK National Joint Registry | Compared to Exeter Stem composite beam and CPT stems affect risk of periprosthetic fracture by a factor 0.4 (95% CI: 0.2-0.7) and 3.9 (95% CI:3.1-4.9) | II |
| *Lamb JN, et al*  Risk Factors for Revision of Polished Taper-Slip Cemented Stems for Periprosthetic Femoral Fracture After Primary Total Hip Replacement: A Registry-Based Cohort Study from the National Joint Registry for England, Wales, Northern Ireland and the Isle of Man.  J Bone Joint Surg Am. 2020 Sep 16;102(18):1600-1608 | 2020 | UK National Joint Registry | Compared to Stainless steel stems Cobalt Chromium stems increase risk of PFF by a factor of 6.7 (95% CI: 3.0-15.4) | II |
| *Hirata M, et al*  Relationship between the Surface Roughness of Material and Bone Cement: An Increased "Polished" Stem May Result in the Excessive Taper-Slip.  Materials (Basel). 2021 Jul 2;14(13):3702 | 2021 | Lab study | Cobalt-Chromium has significantly low coefficient of friction with cement that stainless stell | II |
| *Baryeh K, et al*  Temporal subsidence patterns of cemented polished taper-slip stems: a systematic review. EFORT Open Rev. 2021 May 4;6(5):331-342 | 2021 | Systematic Review | CPT (Cobalt Chromium) stems subside at greater rates than stainless-steal stems (0.37mm/yr vs 0.1-0.15mm/yr) | II |
| *Baryeh K et al. Periprosthetic femoral fractures around the original cemented polished triple-tapered C-stem femoral implant: a consecutive series of 500 primary total hip arthroplasties with an average follow-up of 15 years. Arch Orthop Trauma Surg. 2022 Nov 29:1–8.* | 2022 | UK Single centre | “A PFF rate of 1.4% at an average follow-up of 15 years represents the true incidence of PFF with the use of the original triple-tapered C-Stem femoral implant, similar to that of published Exeter series (1.85%) but lower than the CPT (3.3%)” |  |
| **Meta-analyses: Open Reduction Internal Fixation Vs Revision for PFF** | | | | |
| *González-Martín et al*  Osteosynthesis versus revision arthroplasty in Vancouver B2 periprosthetic hip fractures: a systematic review and meta-analysis. Eur J Trauma Emerg Surg. 2022 Jul 5 | 2022 | Systematic Review | ORIF compared with revision arthroplasty has a shorter surgical time, less need for blood transfusion, fewer complications and reoperation rate and shorter hospital stay; with similar functional outcomes and first-year mortality | II |
| *Lewis DP, et al.*  Management of Vancouver B2 Periprosthetic Femoral Fractures, Revision Total Hip Arthroplasty Versus Open Reduction and Internal Fixation: A Systematic Review and Meta-Analysis. J Orthop Trauma. 2022 Jan 1;36(1):7-16 | 2022 | Systematic Review | ORIF was associated with a lower dislocation rate but complication rate between ORIF and revision was otherwise equivalent. | II |
| *Haider T, et al*  Revision Arthroplasty Versus Open Reduction and Internal Fixation of Vancouver Type-B2 and B3 Periprosthetic Femoral Fractures. JBJS Rev. 2021 Aug 20;9(8) | 2021 | Systematic Review | No difference was observed between ORIF and revision with regard to clinical or radiographic outcome or complication rate | II |
| *Stoffel et al.*  Fracture fixation versus revision arthroplasty in Vancouver type B2 and B3 periprosthetic femoral fractures: a systematic review.  Arch Orthop Trauma Surg. 2020;140(10):1381-1394 | 2020 | Systematic Review | “ORIF may be a viable option if bone stock is adequate around uncemented or tapered polished stems with an intact cement mantle and the fracture geometry allows stable anatomic reconstruction.” | II |
| **Open Reduction Internal Fixation vs Revision for PTS PFF** | | | | |
| *Slullitel PA, et al.*  Selected Vancouver B2 periprosthetic femoral fractures around cemented polished femoral components can be safely treated with osteosynthesis. Bone Joint J. 2021;103-B(7):1222-1230 | 2021 | Buenos Aires, Argentina | “Low-demand…patients with B2 fractures around well-cemented polished femoral components with an intact bone-cement interface can be treated with internal fixation” | III |
| *Powell-Bowns et al.*  Vancouver B periprosthetic fractures involving the Exeter cemented stem. Bone Joint J. 2021;103-B(2):309-320. | 2021 | Edinburgh, UK | “When the bone-cement interface was intact and the fracture anatomically reducible, all Vancouver B fractures around Exeter stems could be managed with fixation. Fixation is associated with reduced need for blood transfusion and lower risk of revision surgery compared with revision.” | III |
| *Smitham PJ, et al.*  Vancouver B2 peri-prosthetic fractures in cemented femoral implants can be treated with open reduction and internal fixation alone without revision.  J Arthroplasty. 2019;34:1430–1434 |  | Australia/New Zealand/South Korea | In 52 patients of median age with B2 PFF treated with ORIF Harris Hip Scores were favourable with non-requiring stem revision. | III |
| *Jain S, et al*  A multicentre comparative analysis of fixation versus revision surgery for periprosthetic femoral fractures following total hip arthroplasty with a cemented polished taper-slip femoral component. Bone Joint J. 2023 Feb;105-B(2):124-134. | 2023 | UK | Revision surgery similar reoperation rates, longer surgical waiting times, higher transfusion requirements, and higher critical care requirements than ORIF in the management of B2/B3 periprosthetic fractures around polished taper-slip femoral components after THA. ORIF is a safe option providing anatomical reconstruction is achievable | III |
| **Efficacy of Cement-in-Cement Revision** | | | | |
| *Briant-Evans TW et al*  Cement-in-cement stem revision for Vancouver type B periprosthetic femoral fractures after total hip arthroplasty. A 3-year follow-up of 23 cases.  Acta Orthop. 2009;80(5):548-552*.* | 2009 | Portsmouth, UK | Vancouver B2 PFF can be treated with Cement-in-cement revision in anatomically reducible periprosthetic fractures with a well-preserved pre-existing cement mantle. | III |
| *Maggs JL, et al B2 or not B2?*  That is the question: a review of periprosthetic fractures around cemented taper-slip femoral components. Bone Joint J. 2021 Jan;103-B(1):71-78 | 2021 | Exeter, UK  Brisbane, Australia | Fractures around taper-slip stems with intact cement-bone mantle (B2W) fractures can reliably be managed with cement-in-cement revision | III |
| *Klasan A, et al*  Comparable outcomes of in-cement revision and uncemented modular stem revision for Vancouver B2 periprosthetic femoral fracture at 5 years.  Arch Orthop Trauma Surg. 2022 Jun;142(6):1039-1046. | 2022 | New Zealand, Austria | There was no difference in patient nor implant survivorship in case of CiC revision and uncemented full revision in B2 PFF. However surgical time was shorter with CiC. | III |
